# Dual-echo fMRI can detect activations in inferior temporal lobe during intelligible speech comprehension

**DOI:** 10.1016/j.neuroimage.2015.05.067

**Published:** 2015-11-15

**Authors:** Ajay D. Halai, Laura M. Parkes, Stephen R. Welbourne

**Affiliations:** a Neuroscience and Aphasia Research Unit, School of Psychological Sciences, University of Manchester, Zochonis Building, Brunswick Street, Manchester M13 9PL, UK; b Centre for Imaging Sciences, Institute of Population Health, University of Manchester, Stopford Building, Oxford Road, Manchester M13 9PL, UK

**Keywords:** Dual gradient-echo, Intelligible speech, Inferior temporal lobe, Magnetic susceptibility

## Abstract

The neural basis of speech comprehension has been investigated intensively during the past few decades. Incoming auditory signals are analysed for speech-like patterns and meaningful information can be extracted by mapping these sounds onto stored semantic representations. Investigation into the neural basis of speech comprehension has largely focused on the temporal lobe, in particular the superior and posterior regions. The ventral anterior temporal lobe (vATL), which includes the inferior temporal gyrus (ITG) and temporal fusiform gyrus (TFG) is consistently omitted in fMRI studies. In contrast, PET studies have shown the involvement of these ventral temporal regions. One crucial factor is the signal loss experienced using conventional echo planar imaging (EPI) for fMRI, at tissue interfaces such as the vATL. One method to overcome this signal loss is to employ a dual-echo EPI technique. The aim of this study was to use intelligible and unintelligible (spectrally rotated) sentences to determine if the vATL could be detected during a passive speech comprehension task using a dual-echo acquisition. A whole brain analysis for an intelligibility contrast showed bilateral superior temporal lobe activations and a cluster of activation within the left vATL. Converging evidence implicates the same ventral temporal regions during semantic processing tasks, which include language processing. The specific role of the ventral temporal region during intelligible speech processing cannot be determined from this data alone, but the converging evidence from PET, MEG, TMS and neuropsychology strongly suggest that it contains the stored semantic representations, which are activated by the speech decoding process.

## Highlights

• Intelligible speech comprehension activates bilateral superior temporal lobe.
• Critically, intelligibility also activates anterior inferior temporal lobe.
• Phonological processing does not activate inferior temporal lobe.
• fMRI results converge with existing PET, MEG and neuropsychology.
• Anterior inferior temporal lobe is involved during speech comprehension.

## Introduction

Speech comprehension can be defined as extracting and understanding meaning from incoming auditory signals. In order to understand these signals, one must map them on to stored semantic representations to obtain meaning. There is a large literature on semantic processing, in which these stored semantic representations are shown to be situated bilaterally in the anterior temporal lobes (ATL).These representations are modality and time invariant (Lambon-Ralph et al., 2010, Lambon Ralph et al., 2009, Patterson et al., 2007, Rogers et al., 2004) and therefore might be expected to be the endpoint of the mapping of incoming speech signals. An important piece of evidence comes from the neuropsychological literature of semantic dementia, where patients have multimodal semantic impairments (including comprehension of spoken and written words, pictures, sounds, smell, taste and touch) with preserved episodic memory, perceptual and syntactic processing as well as other aspects of higher order cognition (Bozeat et al., 2000, Coccia et al., 2004, Hodges et al., 1992, Lambon Ralph et al., 1998, Lambon Ralph and Howard, 2000, Lambon Ralph and Patterson, 2008, Luzzi et al., 2007, Patterson et al., 2007, Piwnica-Worms et al., 2010). Converging evidence for the role of ATL in semantic processing arises from studies using TMS (Pobric et al., 2007, Pobric et al., 2010), MEG (Marinkovic et al., 2003), and PET (Mummery et al., 2000, Tranel et al., 2005, Vandenberghe et al., 1996). Importantly, PET imaging studies have shown activation of the ventral ATL, including the inferior temporal gyrus and temporal fusiform gyrus, for speech comprehension in neurologically intact volunteers (Crinion et al., 2003, Scott et al., 2006, Sharp et al., 2004, Spitsyna et al., 2006). Despite this, the lack of evidence of ATL involvement from fMRI investigations appears to have biassed current models of speech processing into omitting the ventral ATL. Instead the speech comprehension literature focuses on the anterior superior temporal sulcus (Rauschecker and Scott, 2009, Scott et al., 2000), posterior temporal gyrus (Hickok and Poeppel, 2007, Okada et al., 2010), anterior middle temporal gyrus (Davis and Johnsrude, 2003) or bilateral STS (Obleser and Kotz, 2010).

A recent meta-analysis attempts to reconcile the variation seen across speech comprehension studies (Adank, 2012). The author identifies 58 experiments (of which 3 where PET and the rest fMRI) that investigated speech comprehension (words, sentences, narratives). The results showed, independent of the design choices, bilateral superior temporal lobe regions, albeit with larger clusters in the left hemisphere as the core network. It was noted that closely matched control items and a sparse scanning procedure were more likely to activate anterior STS regions, whereas sentence or narrative paradigms were more likely to activate left IFG regions. Despite the effect of these design choices, the meta-analysis failed to identify the ventral anterior temporal lobe (vATL) as a key component, which includes the inferior temporal gyrus (ITG) and temporal fusiform gyrus (TFG). Six studies did find activity in left posterior portion of the TFG (Bozic et al., 2010, Davis et al., 2011, LoCasto et al., 2004, Okada et al., 2010, Orfanidou et al., 2006, Rodd et al., 2005); however, this was considerably posterior to the activity reported in the PET imaging literature (Crinion et al., 2003, Sharp et al., 2004, Spitsyna et al., 2006). The absence of the anterior portions of the vATL is likely to be due to the dominance of fMRI studies within the analysis (54 vs. 3 PET), where the reliability of detecting blood oxygenation level dependent (BOLD) changes in anterior vATL regions is reduced (Devlin et al., 2000). In addition, it has been suggested that many previous studies have not used a sufficient field of view to cover the whole brain (Visser et al., 2010).

The fundamental disadvantage of conventional fMRI acquisition protocols is the signal loss experienced at the interface of tissue and fluid/air due to local differences in magnetic susceptibility. The regions most affected by this are the vATL and orbito-frontal gyrus. Devlin et al. (2000) demonstrated the problems of using fMRI to study semantic tasks, by showing that PET was able to detect regions of activation in vATL regions, whereas fMRI could not. One possible alternative to the conventional echo planar imaging (EPI) acquisition used for fMRI is to use a multi-echo technique to overcome magnetic susceptibility problems (Poser and Norris, 2009, Poser et al., 2006). The strategy is to use more than one EPI readouts, one with a short echo time that reduces signal loss in regions with local differences in magnetic susceptibility but at a cost of BOLD sensitivity, and one with a longer echo time to retain superior BOLD sensitivity in the rest of the brain. The combination of these images at short and long echo times provides superior detection power in semantic processing regions (e.g. vATL regions), in comparison to the short and long echo images alone during a visual semantic categorisation task (Halai et al., 2014). Furthermore, the dual echo method outperformed a spin-echo protocol, which has been recently used to detect activity within the vATL during semantic tasks (Binney et al., 2010, Embleton et al., 2010, Visser et al., 2010, Visser and Lambon-Ralph, 2011).

The aim of the current study was to attempt to detect ventral temporal lobe activity using dual echo fMRI during a passive speech comprehension task. This would reconcile the discrepancies between the existing fMRI and PET literatures, and form a consistent picture with converging evidence from neuropsychology.

## Methods

### Subjects

Twenty participants (mean age = 23 years, SD = 5.4 years, 10 males) took part in the study. All subjects were right handed, scoring at least 85 in the Edinburgh Inventory (Oldfield, 1971), native English speakers and had no known hearing problems. The research was approved by a local National Health Service (NHS) ethics committee.

### Experimental paradigm

E-PRIME software was used for the presentation of the stimuli. The participants were asked to passively listen to audio clips and were told that they would be asked questions about what they heard. All subjects were given a practice session before entering the scanner, where they listened to five trials from each condition (not used in experimental run). To avoid confounding visual contamination, we asked participants to close their eyes during the experiment. We used three audio conditions; normal intelligible speech (SP), rotated speech (RS) and rotated vocoded speech (RV) kindly provided to us by Scott et al. (2000). This previous study had also included a noise vocoded condition, which we omitted as participants reported these stimuli to be unintelligible over the scanner noise. Our paradigm is therefore not a direct replication of the previous work although it still allows us to construct contrasts for intelligibility and phonology. The sentences have a simple ‘subject-verb-object’ structure, and were matched for complexity. The experiment consisted of 90 blocks lasting 15 s each, with 15 blocks per condition separated by a 15 s rest block (only scanner noise). Each experimental block contained 5 items, where each item lasted 2000 ms, and was prefixed with a random ISI from a pool of 800, 900, 1000, 1100, 1200 ms (maintaining the overall length of the block at 15 s). Each block consisted of items from one condition and the order of blocks was pseudo-randomised using Optseq (http://surfer.nmr.mgh.harvard.edu/optseq/). The experiment lasted 22.5 min split into five 4.5 minute runs to help the participant avoid tiredness. The auditory stimuli were delivered using a MR compatible noise-cancelling headphone unit developed by MR Confon (http://www.mr-confon.de/).

Once the experimental session was completed, all subjects were given a written two-alternative forced choice task to probe whether they had been paying attention to the sentences. Each sentence from the scanning session was matched with a novel foil sentence that had the same structure as the targets. The sentences were presented above and below a central fixation cross, and subjects had to press a button indicating whether they remembered the top/bottom sentence.

### Image acquisition

All imaging was performed on a 3 T Philips Achieva scanner using an eight-element head coil with a SENSE factor of 2.5. The continuous dual-echo sequence included 42 slices covering the whole brain with a short and long TE of 12 ms and 35 ms, TR = 3.7 s, 96 × 96 acquisition matrix, FOV = 240 × 126 × 240 mm, in-plane resolution 2.5 mm × 2.5 mm, and slice thickness 3 mm (no gap). The experiment consisted of 5 runs each with 78 volumes (390 volumes in total). A high-resolution T_1_-weighted structural scan with in-plane resolution of 0.94 mm and slice thickness of 0.9 mm was also obtained for co-registration purposes. A high-resolution T_1_-weighted structural image was acquired for co-registration purpose, using a 3D MP-RAGE sequence with in-plane resolution of 0.94 mm and slice thickness of 0.9 mm.

### fMRI data analyses

#### Pre-processing

The original images from the scanner were initially analysed using in-house MATLAB code (available upon request). This extracts an image volume for each echo and subsequently combines the short and long echo for each TR using simple linear summation. Subsequent processing and statistical analysis were carried out using statistical parametric mapping (SPM8) software (Wellcome Trust Centre for Neuroimaging). Single subject EPI volumes were corrected for motion artifacts by registering them to the first image volume using a rigid body spatial transformation. The mean functional volume was co-registered to the subjects' T_1_-weighted image. DARTEL (Diffeomorphic Anatomical Registration using Exponentiated Lie algebra; Ashburner, 2007) was used to improve inter-subject registration and precision in anatomical localisation (Klein et al., 2009). The T_1_-weighted image of each subject was partitioned into grey matter, white matter and CSF tissue classes using SPM8's ‘new segment’ toolbox. Following this, we used the DARTEL toolbox to create a group template derived from all of the subjects in our dataset. The grey matter component of this group template was then registered to the SPM8 grey matter probability map (MNI standard stereotactic space), by estimating a 12-parameter affine transform. In the process of creating the template brain, DARTEL also outputs ‘flow fields’ for each subject, which contain the transform from the original T_1_-weighted image space to that of the group template. Each subject's flow field was combined with the group template-to-MNI transform using SPM8 deformation utility. This transformation from subject native space to MNI space was then applied to all functional image volumes, with a smoothing kernel of 8 mm full-width half maximum (FHWM) Gaussian filter and re-sampled to 3 × 3 × 3 mm voxel size using tri-linear interpolation. Functional images were also temporally filtered using a high-pass filter with a cut-off of 128 s (~ 0.0078 Hz). The normalised segmented grey matter image was converted into a binary mask and used as the statistical mask for analysis.

#### Statistical analysis

Data were analysed using the general linear model approach. At the individual subject level, each task was modelled as a boxcar function (resting blocks were modelled implicitly), and subsequently convolved with the canonical hemodynamic response function. Contrasts were calculated to assess activations for speech (SP > RV), intelligibility (SP > RS) and phonology (RS > RV). The whole brain multi-subject analysis was carried out using a random effects model with a one-sample t-test. The group t-map was assessed for cluster-wise significance using a FDR cluster threshold of p = 0.05 based on a voxel height threshold of p = 0.001. In order to understand the relative contribution of the long and short echo, we separately analysed the intelligibility contrast for each echo in whole brain and ROIs within the ventral temporal lobe (8 mm sphere taken from − 42 − 16 − 34 and − 38 − 18 − 32) (Crinion et al., 2003, Sharp et al., 2004, respectively). Signal loss and image distortions can result in missing data within the affected regions, and therefore we examined the effect of masking on the results by considering the data with and without a masking threshold (although see Vaden et al., 2012 for ways to deal with missing data). Furthermore, we calculated the contrast-to-noise ratio (CNR) within two ROIs for the long and short echo data. The ROIs were 8 mm spheres centred at the peak activation in the mSTS and vATL for the intelligibility contrast. The CNR provides a measure of the image quality based on BOLD contrast. There are multiple interpretations in the literature as to the method of calculating CNR (see Welvaert and Rosseel, 2013 for detailed comparisons), however for the current report we used the following formulation: CNR = $\frac{A}{\sigma N}$, where A is the amplitude of the BOLD signal and σN is the standard deviation of the temporal noise within the ROI (definition 2 in Welvaert and Rosseel, 2013).

Finally, using an ROI approach we explored the relative activations for each condition against rest (continuous fMRI scanner noise) to understand the relative profile of auditory processing. Peaks were obtained from Scott et al. (2000), which are located across the left STS in addition to the ventral inferior temporal/fusiform gyrus from the current intelligibility contrast. The right hemisphere peaks were obtained by taking the homologue of the left hemisphere peaks (see Fig. 4; 8 mm spheres at [± 60 − 4 − 10], [± 64 − 38 0], [± 54 6 − 16], [± 66 − 12 − 12], and [− 42 − 12 − 45]).

## Results

### Behavioural analysis

During debriefing all participants said they heard the stimuli, and this was indicated by all subjects scoring above chance in the two-alternative forced choice test. The worst scoring participant correctly identified 42 of the sentences, which is significantly above chance (p = 0.027, one tailed), while the best performing participant identified 61 sentences correctly.

### Whole brain analysis

**Fig. 1:**
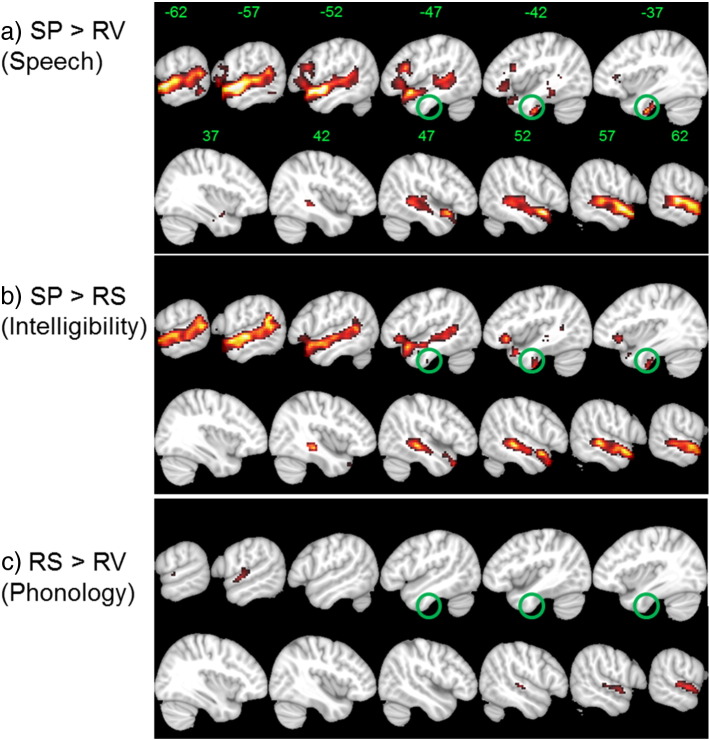
Significant fMRI activations for intelligible and unintelligible sentences: a) speech, b) intelligibility and c) phonology processing. *Activations are FDR cluster corrected at p* = *0.05 based on an uncorrected voxel-threshold of p = 0.001. Sagittal slices are shown centred around the left (negative slices) and right (positive slices) temporal lobes. SP (normal speech); RS (rotated speech); RV (rotated vocoded speech).Scale showing t-values (range 3–8).*

Before group level analysis was carried out, two subjects were removed for excessive movement during the scan (> 5 mm in any direction). The subsequent analysis was conducted on 18 subjects and the figures shown are FDR cluster corrected at p = 0.05 based on a voxel height threshold of p = 0.001. Fig. 1 shows the results for speech (SP > RS), intelligibility (SP > RV) and phonology (RS > RV) contrasts. The speech contrast showed significant activation in the border between the left anterior inferior temporal gyrus (− 42 − 12 − 42) and anterior/posterior fusiform gyrus (− 33 − 12 − 39/− 36 − 18 − 33). Similar activity was observed for the intelligibility contrast, with the peak in the anterior inferior temporal gyrus (− 42 − 12 − 45) extending into the anterior fusiform gyrus. Both contrasts produced activations that spanned from the posterior end of the STG bordering the posterior SMG to the anterior temporal pole, with the highest activations located in the aSTG (− 60 − 3 − 9) and temporal pole (− 50 14 − 17). Both contrasts showed bilateral activity along the superior part of the temporal lobe and the right hemisphere peaks for intelligibility were in the aSTG (63 0 − 9) and TP (54 12 − 8), with an additional peak in pMTG (51 − 30 − 6) (similar for the speech contrast).

There were no significant activations for phonology within the ventral temporal lobe, although it did reveal smaller bilateral STS activity. In the left hemisphere, this was anterior to the primary auditory cortex and located in the planum temporale (− 60 − 12 3) and aSTG (− 57 − 9 0). In the right hemisphere, the activity was largely on the lateral surfaces of the aSTG (60 − 3 − 3) and pSTG (72 − 12 3). The peak activations and cluster sizes are shown in Table 1.

### Region-of-interest (ROI) analysis

**Fig. 2:**
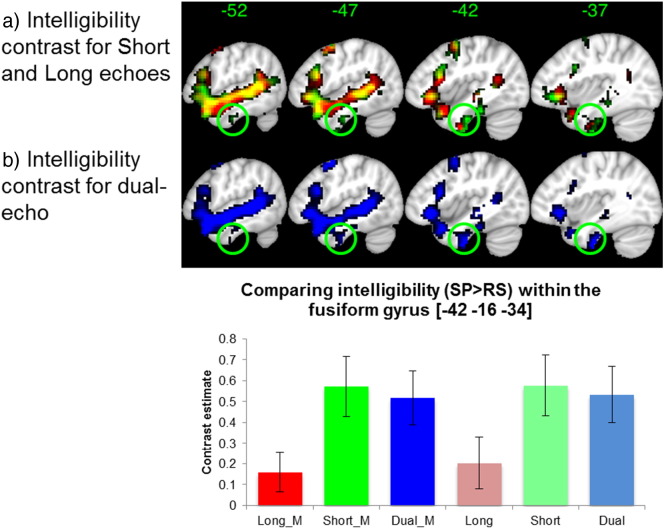
Comparing intelligibility contrast (SP > RS) across the echoes. 2a). Showing results from short (green) and long (red) echoes, with overlap in yellow. The circle highlights the contribution of short echo within inferior temporal regions, compared to long echo. 2b). Showing dual-echo data and the comparison between its constituent parts, highlighting the inferior temporal region. Scale showing t-value range (2–6) for a) and b). The bar graph shows the contrast estimates for intelligibility within an independent regions-of-interest (ROI; 8 mm sphere at − 42 − 16 − 34 (Crinion et al., 2003). The first three columns represent the data with standard SPM masking (suffix _M) and last three columns show the data without masking. The dual and short echo is always significantly larger than the long echo (all p's < 0.025), while there is no difference between the dual and short echoes.

The results from the short echo show that it is driving the detection of activity within vATL regions (Fig. 2). The bottom panel (bar graph) shows contrast estimates for intelligibility (SP > RS) within an independent ROI centred on the anterior fusiform gyrus (− 33 − 3 − 3, Crinion et al., 2003). The result highlights a significant increase, in favour of the dual echo within this region compared to the long echo (t(1,17) = 3.31, p = 0.004). In addition, we performed the same analysis for the peak ROI in Sharp et al., 2004 and found the same results, where the dual echo performed better than the long echo (t(1,17) = 2.90, p = 0.01). In order to understand the effect of missing data, we reanalysed the data removing the masking threshold. Fig. 2 (bottom panel, right three columns) shows the data without masking, which shows the same result as with masking (left three columns). We quantified the percentage overlap between the independent ROI and the group statistical mask with and without applying the default mask threshold. When there is no threshold on the masking, the ROI completely covers the group statistical mask for all echoes (100%). The same data with the default-masking threshold in SPM8 results in the long echo overlapping only 20% with the ROI, compared to 72% using the dual echo.

**Fig. 3:**
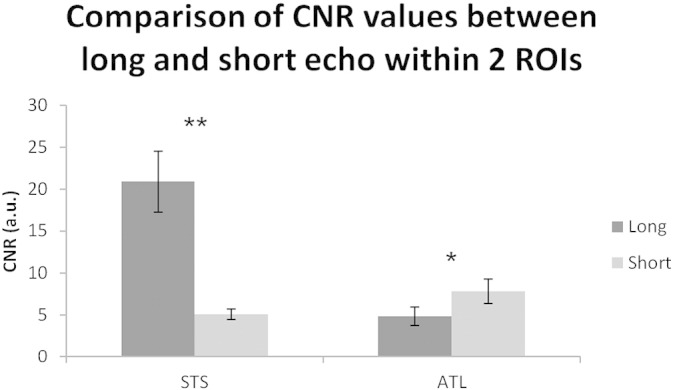
Contrast-to-noise ratio for short and long echo within two ROIs: mid superior temporal sulcus (STS) and ventral anterior temporal lobe (ATL). The long echo had significantly higher CNR in the STS compared to the short echo (**p < 0.001). However, the ATL is in a region affected by magnetic susceptibility artifacts, therefore the short echo showed significantly higher CNR compared to the long echo (*p = 0.046).

It should be noted that in regions not affected by magnetic susceptibility artifacts it is clear that it is the long echo that drives the detection of activity, with t-values along the STS and IFG that are generally greater than those found in the short echo. These findings are expected as the CNR was found to be greater for the long echo in the mid STS region, which is unaffected by magnetic susceptibility compared to the short echo. In contrast, the short echo data showed greater CNR in the vATL region, which is affected by magnetic susceptibility artifacts that are detrimental to the long echo (Fig. 3).

**Fig. 4:**
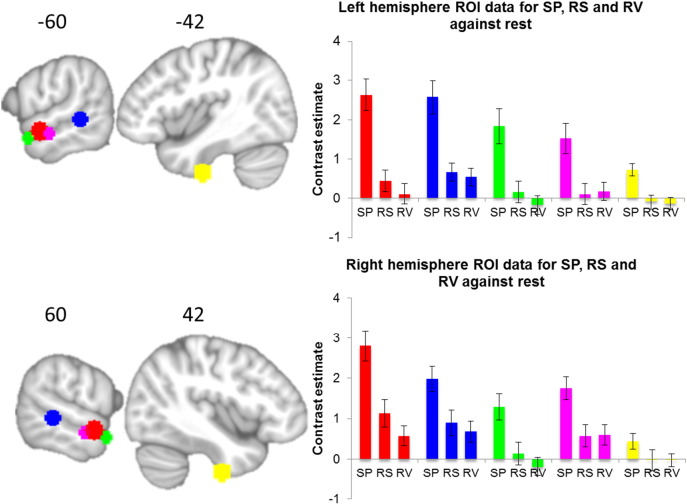
ROI plots for normal speech (SP), rotated speech (RS) and rotated vocoded speech (RV) against rest (continuous fMRI scanner noise) at peaks from Scott et al. (2000) and the ventral inferior temporal gyrus from the intelligibility contrasts (SP > RS) (right hemisphere peaks were taken as the homologue of the left hemisphere). ROIs created using 8 mm spheres at peak coordinates: red [± 60 − 4 − 10], blue [± 64 − 38 0], green [± 54 6 − 16], purple [± 66 − 12 − 12], and yellow [− 42 − 12 − 45].

Finally, we present the data for each stimuli condition against rest at ROIs across the left and right superior temporal lobe and ventral temporal lobe, in order to determine the profile of activity (Fig. 4). The main observation is that the results are bilateral and all ROIs respond highest to normal speech. The two ROIs located posteriorly (red and blue) show some response to RS and RV, however the more anterior the ROI (green and purple) the more specifically the region responded to normal speech only (except the right purple ROI). This is mirrored in the profile of the left and right ventral temporal lobes albeit with lower signal compared to activity along the superior temporal lobe. The lower signal may be due to the loss of the contribution from the long echo in this magnetic susceptible region, which is primarily driven by the short echo only.

## Discussion

The aim of the study was to determine if dual-echo fMRI could detect activity in ventral temporal regions during speech comprehension. The current dual-echo fMRI result provides evidence that bilateral anterior inferior temporal gyrus and temporal fusiform gyrus are involved during auditory semantic processing as would be predicted from consideration of converging PET (Crinion et al., 2003, Scott et al., 2006, Sharp et al., 2004, Spitsyna et al., 2006, Tranel et al., 2005), MEG (Marinkovic et al., 2003) and neuropsychological studies (Hodges et al., 1992, Patterson et al., 2007). Furthermore, it provides additional evidence that the dual-echo protocol is sensitive enough to detect semantic processing activity in the vATL (Halai et al., 2014). We propose that current models of speech comprehension, which omit this region, need to be updated to include the vATL as a core part of the comprehension network (Hickok and Poeppel, 2007, Rauschecker and Scott, 2009).

We believe that this study is the first to show activation in the vATL during passive sentence-level speech comprehension using an fMRI protocol. A recent study using spin-echo fMRI also identified activity within the ventral fusiform region during an active task for auditory words compared to pink/brown noise bursts (Visser and Lambon-Ralph, 2011). In contrast, participants in the current study passively listened to sentence level presentations, which were compared with acoustically matched spectrally rotated speech conditions. This control provides a closer matched condition than pink/brown noise as the rotated speech contains complex acoustic information (formant-like acoustic features) that is not present in pink/brown noise (e.g. Scott et al., 2000). That is not to say that this region is specific for speech processing and the auditory domain. There is considerable converging evidence within the semantic cognition literature that has implicated the vATL during the formation of semantic representations that are both modality and time invariant (Lambon-Ralph et al., 2010, Lambon Ralph et al., 2009, Patterson et al., 2007, Rogers et al., 2004). In other words, regardless of the sensory input (written words, pictures, auditory words or sounds), the ATL enables activation of the same semantic representation. Damage to the ATL regions, as in the case of semantic dementia patients, results in multimodal semantic impairments with preserved episodic memory, perceptual and syntax information, as well as other higher order cognition (Hodges et al., 1992, Lambon Ralph and Patterson, 2008, Patterson et al., 2007). For example, comprehension is impaired for spoken and written words, pictures, sounds, smell, taste, and touch (Bozeat et al., 2000, Bozeat et al., 2003, Coccia et al., 2004, Luzzi et al., 2007, Piwnica-Worms et al., 2010). Recent studies using a novel spin-echo fMRI method have also shown the amodal sensitivity of the ventral anterior temporal region (Binney et al., 2010, Embleton et al., 2010, Hoffman et al., 2015, Visser et al., 2010). Further investigations into the specific role of sub-regions of the vATL have begun to emerge. For example, it has been shown that the left and right vATL respond significantly to pictures, auditory words and environmental sounds, where auditory words respond higher in the left than right vATL (Visser and Lambon-Ralph, 2011). Furthermore, in the same study, the aSTS only responded to both auditory stimuli but not to pictures. This suggests that the aSTS processes aspects of high-order auditory information, which works with the vATL to achieve speech meaning, while the visual information is processed via projections from the occipital to the inferior temporal cortex (Sharp et al., 2004, Visser and Lambon-Ralph, 2011). The vATL might be expected to have the same role in speech comprehension as it does in other semantic tasks, namely providing meaning. Therefore, one would expect activation of vATL during successful comprehension, be it at the word, sentence or discourse level. However, the current models of speech processing do not implicate the vATL as a component and instead focus on aSTS (Rauschecker and Scott, 2009) or pMTG/ITS (Hickok and Poeppel, 2007). This could be largely due to the types of studies used to develop the models, where the fMRI literature has heavily dominated. In contrast, Spitsyna et al. (2006) used PET to outline how the comprehension network can incorporate the vATL from sensory auditory and visual inputs.

The results for the speech (SP > RV), intelligibility (SP > RS) and phonology (RS > RV) contrasts produced bilateral activations on the STS/STG. The peak activations for phonology were located at the PT extending anteriorly, in contrast to the peak activations for intelligibility, which were located in the aSTG and temporal pole. The anterior location of the intelligibility effect provides evidence for a number of neuroimaging studies (McGettigan et al., 2012, Narain et al., 2003, Scott et al., 2000, Scott et al., 2006) and speech comprehension models (Rauschecker and Scott, 2009). In contrast to the left lateralised view of these studies, we found a bilateral network of speech comprehension (Fig. 4), supporting a recent meta-analysis (Adank, 2012). The results showed that the anterior superior temporal gyrus and temporal pole and inferior temporal gyrus responded more selectively to normal speech, in comparison to the posterior superior temporal gyrus, which although responded highly to normal speech, it also responded to acoustically complex unintelligible sounds. This pattern of results does support the hypothesis of a posterior-anterior graded specialisation for speech comprehension (Scott et al., 2000). One possible confounding factor could be related to the use of continuous fMRI scanning as opposed to sparse sampling, which results in high-levels of competing background noise (although this will have been reduced by the use of noise cancelling headphones). Peelle et al., 2010a, Peelle et al., 2010b suggest that background noise introduces additional task components such as increased listening effort. They found that increasing the listening effort resulted in increased BOLD response in left supramarginal gyrus and posterior STS, which is not part of the core comprehension network (Adank, 2012). The results of a recent meta-analysis showed that sparse fMRI scanning promoted activity within bilateral aSTG, left pSTG and left angular gyrus (AG), while continuous fMRI scanning promoted activity within supplementary motor area (SMA) (Adank, 2012). As the current study used continuous fMRI scanning, we cannot completely rule out the possibility that part of the network observed might be due to attentional processes.

The activity in IFG seen in the current data supports the finding that studies using sentence/narratives are more likely to identify left pars opercularis and pars triangularis. It can be argued that these regions are involved in processing longer segments of data, and this could involve syntax or semantic integration (e.g. Newman et al., 2003, Thompson-Schill et al., 1997). The speech comprehension model proposed by Hickok and Poeppel (2007) does not consider the IFG as part of the speech comprehension network (instead as part of the production network), and therefore our results are inconsistent with that suggestion. However, Rauschecker and Scott (2009) propose the IFG to be actively involved during speech comprehension by mapping speech sounds onto abstract forms in the mental lexicon. One possible line of supporting evidence for this idea comes from a recent tractography study that revealed anatomical connections between the anterior temporal pole and the pars orbitalis via the uncinate fasciculus (Binney et al., 2012).

It is noted that a number of methodological factors need to be considered when researchers are interested in studying regions that are affected by magnetic susceptibility artifacts. Indeed, these choices are further constrained by a number of practical factors, for example by the limitations of the scanner, the need to minimise the TR (and so increase the statistical power by increasing the number of images) and the need to maintain whole brain coverage. The dual-echo protocol can increase the TR due to the read-out of an additional echo volume. This is partly scanner dependent—first generation scanners suffered from gradient coil over-heating, which was compensated by longer TRs as in this study. More recent scanners have greatly improved gradient cooling mechanisms, which resolve this problem. TR also increases with higher image resolution so although thinner slices might be beneficial in reducing signal voids (Robinson et al., 2004), it will reduce coverage or reduce SNR (if slice gap is increased to maintain coverage). Reducing the in-plane resolution by using a 64 × 64 acquisition matrix would allow for smaller spacing between the echoes and reduced TR. The parameters used in the current study reflect the upper limits of dual-echo fMRI on a first generation scanner, using a relatively high spatial resolution while maintaining whole brain coverage. Further work is needed to establish how a dual-echo approach compares with modified single echo imaging protocols that are designed to reduce signal loss and distortion. For example, with thinner slices (Robinson et al., 2004) and reduced TE.

In addition to imaging parameters, the design and pre-processing steps can influence the statistical power of the study. For example, the decision to employ an event-related or sparse sampling design reduces statistical power and results in increasing the overall length of these studies. The current study had increased statistical power by employing a block design; however the experimental length was relatively short (22.5 min) compared to similar fMRI studies (typically 30–40 min). The overall length in the current study was constrained by the fact that a passive listening paradigm was used during which participants had their eyes closed. It is likely that longer passive experiments would increase the likelihood of participants falling asleep. In addition to the design parameters, pre-processing steps could affect the outcome at the group level. For example, using a non-linear inter-subject alignment (DARTEL in SPM8) has been shown to transform native space data to normalised space (in this case MNI space) more accurately than using the standard normalisation procedure in SPM8, and indeed many other available normalisation methods (Klein et al., 2009). It is critical to note, that the combination of the imaging parameters, study design and pre-processing steps results in the dual-echo data identifying a significant effect of intelligibility within the vATL. When taking into account each echo separately, it is clear that the short echo is driving the activity within vATL regions, whereas the long echo is driving activity within regions unaffected by magnetic susceptibility artifacts (see Fig. 2, Fig. 3). It suggests that the combination of the two echoes provides the benefit of detecting reliable changes in the BOLD signal across all regions of the brain (Halai et al., 2014, Poser and Norris, 2009, Poser et al., 2006), including the vATL which is increasingly implicated in semantic cognition (Visser et al., 2010).

Finally, it should be noted that an fMRI method that is sensitive to BOLD changes in the ventral temporal region could help better inform brain connectivity analysis. For example, Leff et al. (2008) performed a dynamic causal modelling (DCM) analysis that investigated how three regions involved in intelligible speech comprehension were functionally connected (aSTS, pSTS and IFG). This network of regions does not include the vATL, which is suggested to be critically involved during speech comprehension. In contrast, dual-echo fMRI would increase the likelihood of detecting BOLD changes in ventral temporal regions, which then can be included in analyses, such as DCM to provide a better understanding of the connectivity of the speech comprehension network. Although there are technical difficulties in investigating temporal/effective connectivity using DCM of fMRI data (i.e. outcome biassed by a priori nodes selected, slow temporal change of fMRI signals), it can be a useful exploratory tool.

## Conclusions

The current study proposes the use of a novel dual-echo fMRI approach for investigating the function of the vATL and regions conventionally affected by magnetic susceptibility artifacts. We identified activity within the vATL during passive speech comprehension using a dual-echo approach, highlighting its importance as a repository of semantic representations that should be central to comprehension. Although there is convergent evidence from other modalities for the role of the ventral temporal lobe, further study is needed using modified fMRI techniques that are able to image the susceptible regions reliably if we are to better understand the role of the vATL.

## Figures and Tables

**Table 1:** MNI peaks identified for speech, intelligibility and phonology contrast (sentences). The Harvard–Oxford atlas was used to identify the corresponding anatomical region for each peak. Abbreviations: superior temporal gyrus (STG); middle temporal gyrus (MTG); inferior temporal gyrus (ITG); temporal fusiform gyrus (TFG); inferior frontal gyrus (IFG); orbitofrontal cortex (OFC); temporal pole (TP); planum temporale (PT); supramarginal gyrus (SMG).

Contrast	Cluster size	Anatomical region	t-Value	z-Value	MNI co-ordinate		
x	y	z
Speech (SP > RV)	837	pSTG	10.84	5.86	63	− 21	0
aSTG	9.61	5.55	60	3	− 9
aSTG	9.32	5.48	63	− 6	− 3
TP	8.99	5.38	54	9	− 14
pMTG	7.66	4.97	51	− 30	− 3
1335	aSTG	10.61	5.81	− 54	− 6	− 6
PT	8.86	5.35	− 57	− 18	0
pSTG	8.40	5.21	− 57	− 27	3
pSMG	8.29	5.18	− 60	− 48	12
TP	8.11	5.12	− 50	11	− 14
pSTG	7.81	5.02	− 63	− 36	6
IFG pOp	6.20	4.42	− 45	18	18
pSTG	5.92	4.31	− 48	− 41	6
IFG pTri	5.08	3.91	− 48	27	0
IFG pOp	4.42	3.56	− 54	12	21
pITG	4.35	3.52	− 45	− 36	− 12
IFG pOp	4.31	3.50	− 57	15	6
77	aTFG	7.09	4.77	− 33	− 12	− 39
aITG	6.40	4.51	− 42	− 12	− 42
pTFG	5.56	4.14	− 36	− 18	− 33
55	Amygdala	6.50	4.55	30	0	− 18
Hippocampus	4.26	3.46	32	− 11	− 29
49	Cerebellum	5.05	3.90	21	− 78	− 45
40	Amygdala	4.72	3.72	− 24	0	− 12
Putamen	4.38	3.53	− 24	3	− 3
Intelligibility (SP > RS)	642	pMTG	10.82	5.85	51	− 30	− 6
aSTG	8.30	5.18	63	0	− 9
TP	7.26	4.83	54	12	− 18
TP	5.40	4.07	48	21	− 30
1280	pSTG	8.65	5.29	− 57	− 18	− 3
aSTG	8.12	5.12	− 60	− 3	− 9
pSMG	7.73	5.00	− 60	− 48	12
TP	6.71	4.63	− 50	14	− 17
OFC	6.28	4.46	− 45	27	− 6
pSMG	6.28	4.46	− 57	− 48	24
pMTG	4.41	3.55	− 42	− 30	− 6
IFG pTri	4.23	3.45	− 54	24	6
32	aITG/aTFG	5.20	3.97	− 42	− 12	− 45
Phonology (RS > RV)	137	aSTG	5.54	4.13	60	− 3	− 3
pSTG	5.02	3.88	72	− 12	3
pSTG	4.97	3.85	66	− 18	3
pSTG	4.63	3.67	72	− 24	9
58	PT	4.78	3.76	− 60	− 12	3
PT/aSTG	4.76	3.75	− 57	− 9	0
